# Bayesian Assessment of True Prevalence of Paratuberculosis Infection in Dairy Herds and Their Parity Subgroups

**DOI:** 10.3390/pathogens14090900

**Published:** 2025-09-07

**Authors:** Katalin Veres, Zsolt Lang, László Ózsvári

**Affiliations:** 1 Department of Biostatistics, University of Veterinary Medicine Budapest, 1078 Budapest, Hungary; lang.zsolt@univet.hu; 2 Department of Veterinary Forensics and Economics, University of Veterinary Medicine Budapest, 1078 Budapest, Hungary; ozsvari.laszlo@univet.hu; 3 National Laboratory of Infectious Animal Diseases, Antimicrobial Resistance, Veterinary Public Health and Food Chain Safety, University of Veterinary Medicine, 1078 Budapest, Hungary

**Keywords:** paratuberculosis, John’s disease, dairy cattle, prevalence modelling, Bayesian latent class modelling, true prevalence, parity, historical priors, Bayes factor

## Abstract

Paratuberculosis is a widespread infectious disease in ruminants that leads to significant economic losses in livestock production. In this study, we developed a practical method for predicting the likelihood of the herd-level presence of the infection and estimating its prevalence in subgroups of a dairy herd—specifically, first-time calving cows (primiparous) and those that have calved more than once (multiparous). We fit a Bayesian hierarchical model to cow-level data, incorporating prior knowledge about regional prevalence of infection to improve the accuracy and reliability of the estimates. The model was tested using synthetic data representing six regional scenarios in four countries (Chile, Denmark, Italy, and Hungary). The likelihood that a herd is infected is evaluated using Bayes factors and posterior probability of infection. Both the Bayes factor and the posterior probability of infection classified the simulated herds in accordance with the proportions of infected herds. Summary measures obtained for within-herd true prevalence estimates demonstrated acceptable accuracy. The R and STAN codes of the model are available as an open-access tool. The model can be customized for any region using real local data and prior information. The relationship between true and apparent prevalence is linear and stable and therefore can be estimated well. We found that, in Hungary, the TP/AP ratios were 1.6 and 1.5 for primi- and multiparous cows, respectively.

## 1. Introduction

Paratuberculosis (PTBC), or Johne’s disease, is a chronic infectious condition caused by Mycobacterium avium subspecies paratuberculosis (MAP), which affects ruminants worldwide and poses a significant challenge to animal health and dairy farm productivity [1,2,3,4,5]. Estimating the prevalence of MAP within herds is a key component of both disease surveillance and economic decision making at the herd level.

Typically, MAP infection happens in the early neonatal period by colostrum, bulk colostrum, or through a contaminated environment. The infection progresses slowly and has a long incubation period, with around 95% of infected cattle in subclinical stage and only 5% displaying clinical signs. Both the clinical and subclinical stages of the infection cause substantial economic losses, but the subclinical stage is economically more influential; at this stage, the animals do not show obvious symptoms but the negative impact on production is already present [5]. The resistance to MAP progression increases with age. However, cattle can also become infected at a later age due to high infection pressure. In addition to the age of the cows, genetics is also likely to influence susceptibility to MAP infection [3].

Direct and indirect methods exist to detect MAP infection. Direct methods are expensive and have a long turnaround time. Milk and serum ELISA are indirect methods detecting the presence of antibodies in the sample. Both have low costs and a rapid turnaround time. Milk ELISA is not invasive and can be routinely and conveniently performed [3]. S/P ratios (numerical ELISA test results) are classified into negative and positive categories according to predefined cut-offs. To avoid excessive false positive results, the cut-off of MAP ELISA is calibrated to favor specificity over sensitivity [6]. ELISA specificity is high (98–100%), its main disadvantage is the low sensitivity (15% for milk ELISA) in the early stages of the infection due to the delay in the appearance of antibodies. The sensitivity depends on the stage of the infection, on the stage of lactation, and on the age of the animal, reaching 90% in clinically affected cattle [3].

The latent and slow progression of MAP infection and the low sensitivity of diagnostic tests make the diagnosis challenging, leading to a marked difference between apparent and true prevalence levels. Disease frequency is the base measure serving as a starting point for the choice of appropriate testing method, control measures, and the assessment of the efficiency of the control program. Using crude apparent prevalence in herd health management can lead to poor decisions and to the failure of control programs. Appropriate statistical methods are needed to adjust apparent prevalence calculated from test results to obtain a prevalence estimate reflecting the true, underlying prevalence of a herd.

There has been considerable research interest worldwide in assessing PTBC prevalence at national or regional level. Nielsen and Toft [7] provided a comprehensive review of paratuberculosis prevalence across European farmed animals, focusing largely on regional data. Hierarchical Bayesian models were fitted to clustered PTBC prevalence data, where the true infection status of herds and animals was unobserved latent classes [8,9,10,11,12]. Herd true prevalence (HTP) and conditional within-herd true prevalence (CWHP) were estimated, using the methods originally developed by Hanson et al. [13] and Branscum et al. [14]. McAloon et al. [11] and other authors [8,9,10,12] modified the methodology of Branscum et al. [14] by introducing new notations and estimated PTBC prevalence in several regions.

In practice, prevalence estimation of different contagious diseases at the herd level is related mainly to substantiating freedom from infection [15], ranging from simple hypothesis tests [16,17] to complex Bayesian frameworks [18].

The aim of this study was to present a Bayesian model developed for a single herd, which can be used to estimate the within-herd animal level true prevalence of PTBC infection separately for primiparous and multiparous cows, as well as to infer the infection status of the herd, based on PTBC diagnostic test results and region-specific prior information. Additionally, we aimed to provide an internationally relevant context for our analysis by integrating prior information from diverse regional studies together with region-specific synthetic data.

## 2. Materials and Methods

We used the Bayes factor and the posterior infection probability to predict herd-level PTBC infection [19,20]. Within-herd animal-level prevalence was estimated using the method developed by Veres et al. [21] in the subgroups of primiparous and multiparous cows. We had real-world data available only from Hungary, but we also generated synthetic datasets with the characteristics of regions for which prior information was available in the literature. To demonstrate how the model worked in practice, we used the Hungarian field data together with relevant prior knowledge. A herd was considered infected if it had at least one truly infected cow. Throughout the study, we assumed the use of the IDEXX milk ELISA test. The sensitivity (Se) of the test was assumed to be age-dependent and calculated according to (1). The specificity (Sp) of the test was considered to be 98.6% [22].

### 2.1. Hungarian Data

A nationwide voluntary PTBC testing program started in February 2018 in Hungary, which was set up for dairy farms to provide help for herd managers to reduce the prevalence and the impact of PTBC in their herds. A Bayesian model was fitted to these data in our previous study [21] and the obtained posterior distribution was used as informative prior in this study. The data consisted of the PTBC screening test results of 55,594 cows in 116 large (number of cows ≥ 100) intensive dairy cattle herds, which represented 24.3% of all dairy cows in Hungary. The detailed description of this dataset and the analysis providing the posterior distributions can be found in Veres et al. [21]. The data collected were used for the following purposes: (1) as the basis for the prior distribution; (2) we illustrated our method using data from two real herds selected from the database; (3) we generated synthetic data for other regions from one of the large Hungarian herds in the database, as described below.

### 2.2. Data for Other Regions

We analyzed synthetic datasets mimicking herds in Hungary, Denmark, Southern Italy, Lombardy (Northern Italy), Veneto (Northern Italy), and Chile. To imitate real-world data, we manipulated the Hungarian data so that it corresponded to the prevalence and the average herd size of the regions analyzed (Table 1).

Based on information from the literature, we generated 20 synthetic datasets for each region as follows: from the data of a sufficiently large Hungarian herd, we drew samples with replacement, in accordance with the average herd size reported for the given region. The original ELISA test results were replaced with independently simulated values. The MAP infection status of the herd was randomly generated from a Bernoulli distribution, with the parameter taken from the prior distribution of the region-specific herd true prevalence (HTP, see Table 2 and Table 3). If the herd was randomized to be infected, the infection probabilities of individual cows in parity subgroups were generated according to a Gaussian copula model [21,27] using region- and parity-specific beta prior distributions (see Appendix B.2 for details).

The age-specific sensitivity was calculated for each cow based on the equation

(1) $$logit\left(Se\left(t\right)\right)=a-b\times e^{-ct},\;$$

where a=1.2, b=3, c=0.3, and $logit\left(x\right)=\ln\frac{x}{1-x}$.

We adopted the parameter values in (1) from [28]. The specificity (Sp) of the test was considered to be 98.6% [22]. We calculated the apparent prevalence as the sum of the probability of true positive and false positive test results (A5). Test results of individual cows were drawn from a Bernoulli distribution with the apparent prevalence as parameter. If the herd was classified as uninfected, individual cow test results were drawn from a Bernoulli distribution with parameter 1-Sp.

### 2.3. Statistical Analysis

#### 2.3.1. Inferring the Infection Status of Herds

To infer the infection probability of the herd, we used Bayes factors [20], which is the ratio of the probability of the observed data in one statistical model to the probability of the same data in another model. The Bayes factor quantifies how much more (or less) likely the data are in one model than in another, thus indicating which model provides a better fit to the observed data. The Bayes factor was calculated using the R package bridgesampling [29], version 1.1.2. In our setting, we compared a model assuming the herd was infected with another model assuming the herd was not infected. The posterior probability of the model given the data is $\frac{BF\times \mu_{HTP}}{BF\times \mu_{HTP}+(1-\mu_{HTP})}$, where BF is the Bayes factor, and $\mu_{HTP}$ is the expected value of the prior distribution of HTP.

#### 2.3.2. Estimating the PTBC Infection Prevalence in a Single Herd

If—based on the assessment of the infection status—we assume that the herd *H* is not infected, then no further calculations are needed. If the herd *H* is thought to be infected, we estimate ${CHWP}_{H1}$ and ${CHWP}_{H2}$, the conditional within-herd prevalences of infection of primiparous and multiparous cows, respectively. A Bayesian latent class model, similar to the one presented in Veres et al. [21], adapted to one single herd, was fitted to the herd data to calculate the posterior distributions of ${CHWP}_{H1}$ and ${CHWP}_{H2}$ of herd H. To calculate the estimates, we used prior information: the results of the ELISA tests, the parity, and the age of the cows. Posterior distributions are the Bayesian estimates of the prevalence of PTBC associated with primiparous and multiparous cows in herd H, respectively.

It is relatively easy to calculate the proportion of positive cases (apparent prevalence, AP) from raw data, but in practice, due to false positive and false negative results, the true underlying prevalence is often different. Our model aims to provide an estimate of the true prevalence of infection, considering the limitations of the diagnostic test.

We assume that the true prevalence of infection in a given herd and parity group is influenced by the regional mean prevalence in that parity group, as well as by unobservable factors specific to the herd and parity group. These factors are modeled as normally distributed random effects, assessed by their variances.

The model approximates the posterior probability distributions of the parameters, conditional on the observed data and on the prior distributions of herd true prevalence (HTP), parity group prevalences (${CHWP}_{H1}$ and ${CHWP}_{H2}$), and the variances of the random effects, by means of the Markov Chain Monte Carlo (MCMC) method. For a more technical description and model checks, see Appendix C.

The Bayesian model runs using the rstan package of the statistical software R 4.1.3 [30,31] (https://github.com/VeresKatalin/PTBCprevalence, accessed on 1 September 2025).

#### 2.3.3. Model Runs

The accuracy of our Bayesian model was tested by simulation. We ran the model on the simulated data of 20 herds for each of the six regions (120 herds in total) and examined the proportion of truly infected herds in which the true prevalence fell within the 95% credible interval (CrI) provided by the model. We also calculated the half-width of the CrI.

#### 2.3.4. Prior Information

The model builds on expert opinions or insights from the historical data of the region, in the form of prior distributions. The model uses priors for the proportion of infected herds in the region (HTP), the mean regional within-herd infection prevalence of primiparous and multiparous cows ($\mu_{1}$ and $\mu_{2}$), and for the variances of the herd- and parity-level random effects.

HTP and $\mu_{1}$ and $\mu_{2}$ are modeled by beta distributions. The beta distribution is defined on the interval [0, 1], making it ideal to fit proportions. Its flexibility allows it to express a wide range of prior beliefs. The variances of the random effects are described by inverse gamma priors. The inverse gamma distribution is commonly used as a prior for variance parameters because it ensures positivity and allows control over the expected variability. Its shape can reflect uncertainty or prior knowledge about the spread of random effects.

Priors for Hungary were defined to best fit the posterior distributions of the analyses of the results of a national PTBC screening program [21] (see Table 2). The procedure epi.betabuster [32] in R 4.1.0 package epiR [33] was used to specify the parameters of the beta distributions.

For Denmark, we derived region-specific CWHP_1_ and CWHP_2_ prior distributions from posterior distributions reported in the literature using the procedure epi.betabuster [32] in R 4.1.0 package epiR [33] to specify the parameters of the beta distributions. For the other regions, we derived region-specific prior distributions from prior distributions found in the literature [8,10,12,23] (Appendix A, Table A1). For the variances of the random effects ($\sigma^{2}$, ${\sigma_{1}}^{2}$ and ${\sigma_{2}}^{2}$), we assumed the same prior distributions as for Hungary in each region. The details of the derivations of $\mu_{1}$, $\mu_{2}$ can be found in Appendix B.1. The priors used in the analysis are shown in Table 3.

## 3. Results

### 3.1. Downloadable Application

The model infers the infection status of the herd and estimates the true prevalence for primiparous and multiparous cows from the data of the individual cows in the herd using prior information to incorporate the region’s characteristics. The software inputs data from one individual herd as xlsx file in the format shown in Table 4.

The model offers six regions to choose from or can be run with user-defined prior distributions and custom data.

### 3.2. Model Results on Real World Data

We illustrate the use of the model with three datasets: (1) a Hungarian herd with no positive test results (Herd 1); (2) a sample of 150 cows (approx. 20%) from Herd 1, to demonstrate model performance when the herd is not fully tested; and (3) a typical Hungarian herd (Herd 2). Herd 2 is typical in the sense that the parity and the age distribution of the cows, as well as the herd size, are representative of a large Hungarian dairy herd.

Table 5 and Table 6 summarize the characteristics of Herd 1. There was no ELISA-positive cow in this herd. The posterior probability of infection of the herd is low; the herd is classified as uninfected based on both the Bayes factor and the posterior probability of infection. If the herd is infected, the model estimates low within-herd prevalences.

Table 7 and Table 8 summarize the characteristics of a sample of 150 cows from Herd 1. The posterior probability of infection of the herd is moderate; the herd is classified as uninfected according to the Bayes factor, and the level of the posterior probability of infection indicates that infection is weakly refuted. If the herd is assumed to be infected, then the model estimates low within-herd prevalences, with wide CrI and different estimates.

Table 9 and Table 10 summarize the characteristics of Herd 2. This is a Hungarian herd with a typical infection prevalence and herd size. The posterior probability of infection of the herd is 100%; the herd is classified as infected based on both the Bayes factor and the posterior probability of infection. The model estimates of true prevalences are 1.6 and 1.5 times greater than the apparent prevalence in the two-parity groups, respectively. These results are consistent with the rule of thumb outlined in Section 3.4.

### 3.3. Results of the Model on Synthetic Data

The model was run on the simulated data of 20 pseudo-herds per region. Table 11 summarizes the results.

We evaluated the performance of the Bayes factor to infer the infection status of the herd. The Bayes factor is not related to the HTP prior of the region. The findings are summarized in Table 12.

We calculated the posterior probability of the infection of the herd. We chose a categorization with five cut points. The findings are summarized in Table 13.

### 3.4. Estimating the PTBC Infection Prevalence in a Single Herd Without the Bayesian Model

The same model was run for all herds tested in the Hungarian national screening program and a linear regression was fitted to the results. The goal was to find a simple relationship linking apparent and true prevalence. The estimated true prevalence was considered as a dependent variable and the apparent prevalence estimated from raw data was considered as an explanatory variable. The intercept was close to zero for both parity groups, so the apparent prevalence multiplied by a simple factor could be used to obtain an estimate of the true prevalence. The true prevalence was estimated as 1.6 times the apparent prevalence for primiparous cows and 1.5 times for multiparous cows in Hungary [34].

## 4. Discussion

This study presents a Bayesian model designed to estimate the true prevalence of paratuberculosis (PTBC) at herd level, separately for primiparous and multiparous cows, by incorporating informative priors derived from regional surveys or reflecting local expert opinions. This approach is particularly suitable for dairy cattle herds in regions where PTBC infection is endemic. Additionally, the model estimates the probability and odds that a given herd is infected by the posterior probability of infection and the Bayes factor, respectively.

Parity, used as a subgroup in the model, is a natural categorization of cows in herd management. This stratification enhances the model’s relevance for practical herd-level decision making. Since primiparous and multiparous cows differ significantly in their susceptibility to infection and infection detectability [6,22,35], handling them together can lead to biased conclusions. Breaking down the herd by parity results in more homogeneous groups with markedly different infection prevalences.

One of the main advantages of the Bayesian approach is that, by incorporating regional priors, the model can consider region-specific factors that cannot be directly measured or quantified. Thus, estimates are based not only on data from the target herd, but also on broader epidemiological patterns and environmental influences characteristic of the region.

In a previous Hungarian study [21], the mean true within-herd prevalence (CWHP) was estimated at 8.4% (95% credible interval: 6.6–10.4%) for primiparous cows and 15.8% (13.5–18.4%) for multiparous cows. Median CWHP estimates were 4.7% (3.2–6.4%) for primiparous and 12.4% (10–15%) for multiparous cows. These values formed the empirical basis for the priors used in our Bayesian model, so the analysis was based on well-characterized national data [21]. To provide an internationally relevant context for our analysis, we integrated prior information from diverse regional studies along with region-specific synthetic data. This approach demonstrated the flexibility and applicability of the model across different contexts.

Ideally, prior distributions are informed by posterior results from previous studies. Hungary and Denmark have published posterior distributions from PTBC prevalence studies using milk ELISA test [21,23]. For these two countries, posterior HTP and CWHP distributions were directly used as prior along with the age-dependent sensitivity and constant specificity values from Veres et al. [21]. For the other four regions, where only serum ELISA test results were available, HTP and CWHP priors were derived from published expert elicitation [8,10,12,23]. Consequently, the resulting posterior distributions may suggest bigger uncertainty than observed in the reality.

For the variance of the random herd effect and additive random parity effects, we initially derived priors from region-specific expert elicitation, but this led to unrealistically high variance estimates and consequent shrinkage toward the mean in some regions. To ensure greater accuracy in the estimates, we used the posterior distribution obtained from the Hungarian study as the prior distribution. While we acknowledge this is a limitation of our approach, we note that assuming the same prior of the variance of the random effects does not imply the same posterior variance of the within-herd animal level prevalence across all regions. The posterior variance of the CWHP also depends on the regional mean of CWHP and the observed data of the target herd.

We applied an age-dependent fixed value for the sensitivity of the diagnostic test, together with a fixed value for its specificity. Using prior distributions instead of fixed values may result in unstable or biased estimates and may lead to an unidentifiable model. The use of fixed values is confirmed by our previous study [21], in which we presented the possible range of Se and Sp, based on available published values and a detailed sensitivity analysis.

In the analysis of the infection status based on the value of Bayes factor, two uninfected herds were classified as “strongly refuted”. Both infected and uninfected herds appeared in the “refuted” category. All herds rated as “supported” and “strongly supported” were indeed infected. For 42 herds, the results were “weakly refuted” or “weakly supported”. Overall, the infection status was correctly identified in 66 of the 78 pseudo-herds that were not classified as weakly refuted or weakly supported.

When estimating the posterior probability of infection of the herds, prior information on herd true prevalence was also taken into account. Out of 120 pseudo-herds, 53 truly infected herds were classified as “strongly supported”, with no herds falling into the “strongly refuted” category. Only one truly infected herd was misclassified into the “refuted” group. The results were “weakly refuted” or “weakly supported” for 48 herds. Overall, the infection status was correctly identified for 69 out of the 72 pseudo-herds with more explicit results. From a Bayesian perspective, misclassification occurs because probabilities should preferably be interpreted as indicators rather than used as classifiers. Errors arise from forcing clear-cut decisions that are fundamentally based on probabilistic information.

Indeterminate classification may arise from a low prevalence in a high herd prevalence region or from a small sample size. If the herd with weak results was not fully tested, then herd managers may try to test a larger sample of cows. In case of a low prevalence in a high herd prevalence region, we recommend evaluating the effectiveness of herd management practices in preventing the spread of the disease between herds. If the practices introduced are appropriate, the herd can be considered uninfected; otherwise, it is reasonable to assume that the herd is infected. To assess the potential level of infection, the estimated CWHP prevalences need to be evaluated along with the width of the credible intervals. This approach provides a comprehensive picture of the infection status of the herd. To confirm the results obtained or to observe changes in the infection status of the herd, we recommend retesting the animals after a certain period of time and analyzing the test results by fitting the presented Bayesian model using the updated, most recent prior distributions. Given the economic and health damage caused by PTBC [4], we believe that it is worthwhile to take measures to prevent and reduce infection even if the farm has been classified as “weakly refuted”.

Table 11 contains summary measures obtained for within-herd true prevalence estimates in truly infected herds. For primiparous cows, the coverage, i.e., the proportion of herds where the simulated within-herd prevalence fell within the CrI, was at least 95%, demonstrating the accuracy of the Bayesian model and the prior distributions of primiparous CWHP in all regions. For multiparous cows the coverage was less accurate in Lombardy (85%) and Veneto (71%). This may indicate that there is too much heterogeneity in the prior distribution of multiparous CWHP in these regions. The mean half length of the CrI measures the uncertainty of estimates. In all regions, it is of the same order of magnitude as the mean primiparous CWHP values, and it is slightly lower than the mean multiparous CWHP values, which seems acceptable from a practical point of view.

In a previous study using Hungarian real-world data, a simple rule of thumb for estimating the true herd-level prevalence has been developed [34]. The model was run on the individual PTBC ELISA milk test results from 116 dairy cattle farms in Hungary. We fitted a linear regression with the estimated true within-herd prevalence as the dependent variable and the apparent prevalence as the explanatory variable. In the regression equation, the intercept estimate was close to zero, so the true within-herd prevalence could be estimated by multiplying the apparent prevalence by a constant [34]. Apart from providing a rule of thumb for Hungary, this method may allow us to derive a similar rule of thumb for other regions using their own herd-level data. It is theoretically appropriate to apply linear regression to pairs of apparent prevalence and estimated true prevalence. According to the relationship between apparent and true prevalences (A5), for specificities close to 1, the ratio of apparent to true prevalence depends primarily on the sensitivity of the test. To investigate the ratio of apparent to true prevalence, we also need to consider the age dependence of sensitivity (1). In Hungarian data, the age heterogeneity of primiparous cows is low, so the sensitivity in this category is relatively stable. Although the age difference is greater in multiparous cows, the sensitivity at higher ages is less dependent on age differences. Thus, for a region with the above characteristics, the relationship between true and apparent prevalence is linear and stable and therefore can be well estimated. The TP/AP ratios of 1.6 and 1.5 that we found for primi- and multiparous cows in Hungary are comparable to the ratios of 1.4, 1.5, and 1.5 published in the Danish study by Verdugo et al. [23] for three consecutive years: 2011, 2012, and 2013. As these ratios depend only on the age distribution and the test sensitivity, for regions using the same milk ELISA test and having similar age distribution to the Hungarian herds, the same values of 1.5 and 1.6 apply.

We note that the model uses synthetic datasets to illustrate the approach, and therefore, regional prevalence estimates should not be interpreted as measures of PTBC prevalence in these areas. When generating synthetic data, we undertook a pragmatic approach: resampling a Hungarian herd and replacing test results to reproduce regional herd sizes and prevalences. This technique may not fully capture the unique characteristics of herds in other regions, such as different age distributions, genetic backgrounds, or management practices, all of which can influence the age-sensitivity relationship and the true prevalence. The results for non-Hungarian regions demonstrate the flexibility of the model rather than its accuracy in these specific locations, where no local validation has taken place. The model can be customized for any region using real local data and prior information.

## 5. Conclusions

The Bayesian latent class model introduced in this study provides estimates of the herd-level infection status and the true prevalence of primiparous and multiparous cows within a single herd. Accurate estimation of within-herd infection prevalence is crucial, as higher prevalence leads to greater production losses and increased culling rates. Infection status assessment and prevalence estimation are the basis for developing effective control and eradication strategies, as well as for monitoring the success of these interventions over time. We demonstrated our model’s potential utility for prevalence estimation and infection status inference across diverse international settings. Combined with high-quality local data and prior information, our approach serves as a robust and adaptable framework to support evidence-based decision making at the herd level. The novelty of the method lies in the estimation of both the probability and the Bayes factor of infection of the target herd, combined with the estimation of infection prevalence within homogeneous subgroups of the herd, specifically parity groups. The approach can be readily generalized to other types of subgroups, such as different breeds, lactation stages, housing pens, or health status.

## Figures and Tables

**Table 1:** Average size of dairy cattle herds reported in the literature and the size of herds used in the region-specific synthetic data.

Region	Average Herd Size ^1^	Herd Size (Synthetic Data)	Source
Denmark	195, 205, 208 ^2^	200	[23]
Southern Italy	315	300	[8]
Lombardy	191	200	[24]
Veneto	100	100	[25]
Chile	83	100	[26]

^1^ Large variability. ^2^ Data from three consecutive years.

**Table 2:** Priors used along with Hungarian data.

Variable	Prior	Median (95th Percentile)
**HTP**	beta (150.589, 12.250)	0.927 (0.955)
$\mu_{1}$	beta (65.700, 715.900)	0.084 (0.101)
$\mu_{2}$	beta (134.195, 716.181)	0.158 (0.179)
$\sigma^{2}$	inverse gamma (37.030, 4.620)	0.126 (0.167)
${\sigma_{1}}^{2}$	inverse gamma (5.330, 0.070)	0.014 (0.032)
${\sigma_{2}}^{2}$	inverse gamma (6.050, 0.090)	0.016 (0.034)

HTP—herd true prevalence; $\mu_{1}$—mean conditional within-herd prevalence for primiparous cows in the region; $\mu_{2}$—mean conditional within-herd prevalence for multiparous cows in the region; $\sigma^{2}$—variance of the herd random effect; ${\sigma_{1}}^{2}$—variance of the parity random effect in primiparous cows; ${\sigma_{2}}^{2}$—variance of the parity random effect in multiparous cows.

**Table 3:** Priors used in the study.

Region	Parameter	Priors and Parameters Used in the Analysis	Median (95th Percentile)
Denmark	HTP	beta (425.110, 144.641)	0.746 (0.756)
	$\mu_{1}$	beta (1.770, 43.210)	0.033 (0.095)
	$\mu_{2}$	beta (3.850, 45.090)	0.073 (0.150)
Southern Italy	HTP	beta (5.030, 7.040)	0.412 (0.650)
	$\mu_{1}$	beta (2.766, 46.335)	0.050 (0.118)
	$\mu_{2}$	beta (6.018, 47.403)	0.108 (0.019)
Northern Italy (Lombardy, Veneto)	HTP	beta (13.320, 6.280)	0.686 (0.838)
	$\mu_{1}$	beta (1.079, 18.347)	0.041 (0.157)
	$\mu_{2}$	beta (2.347, 18.788)	0.099 (0.239)
Chile	HTP	beta (14.200, 0.700)	0.971 (0.999)
	$\mu_{1}$	beta (12.896, 172.151)	0.068 (0.103)
	$\mu_{2}$	beta (28.061, 173.629)	0.138 (0.181)

HTP—herd true prevalence; $\mu_{1}$—mean conditional within-herd prevalence for primiparous cows in the region; $\mu_{2}$—mean conditional within-herd prevalence for multiparous cows in the region.

**Table 4:** Input data format for the model. HERD_ID: integer, herd ID. COW_ID: cow ID, integer, unique. MULTIPAR: parity indicator—0 for primiparous cows; 1 for multiparous cows. POS: milk ELISA positivity indicator—1 for test-positive cows; 0 for test-negative cows.

HERD_ID	COW_ID	MULTIPAR	AGE	POS
1000	1001	0	2.7186202	0
1000	1002	0	2.1694455	0
1000	1003	0	2.3301748	0
1000	1004	0	2.4988055	0

**Table 5:** Descriptive characteristics and true prevalence estimates (Herd 1).

Subgroup	Number	Number of Positives	Apparent Prevalence	Estimated True Prevalence (95% CrI)
Primiparous cows	331	0	0%	0.1% (0;0.4)
Multiparous cows	390	0	0%	0.4% (0;1.4)
Overall	721	0	0%	

**Table 6:** Bayes factor and posterior probability of the herd being infected (Herd 1).

Measure	Value	Decision
Bayes factor	0.016	infection strongly refuted
Posterior probability	17.54%	infection refuted

The decision rules are based on Kass and Raftery [20].

**Table 7:** Descriptive characteristics and true prevalence estimates for a sample of 150 cows from Herd 1.

Subgroup	Number	Number of Positives	Apparent Prevalence	Estimated True Prevalence (95% CrI)
Primiparous cows	61	0	0%	0.4% (0;2.4)
Multiparous cows	89	0	0%	1.4% (0.1;5.6)
Overall	150	0	0%	

**Table 8:** Bayes factor and posterior probability of the herd being infected for a sample of 150 cows from Herd 1.

Measure	Value	Decision
Bayes factor	0.071	infection refuted
Posterior probability	47.42%	infection weakly refuted

The decision rules are based on Kass and Raftery [20].

**Table 9:** Descriptive characteristics and true prevalence estimates (Herd 2).

Subgroup	Number	Number of Positives	Apparent Prevalence	Estimated True Prevalence (95% CrI)
Primiparous cows	205	12	5.9%	9.3% (4;15.9)
Multiparous cows	262	31	11.8%	17.7% (12.1;24.2)
Overall	467	43	9.2%	

**Table 10:** Bayes factor and posterior probability of the herd being infected (Herd 2).

Measure	Value	Decision
Bayes factor	6.17 × 10^20^	infection strongly supported
Posterior probability	100%	infection strongly supported

The decision rules are based on Kass and Raftery [20].

**Table 11:** Summary measures characterizing the model estimates for the true prevalence in truly infected herds.

	Primiparous Cows			Multiparous Cows		
Country	Coverage of 95% CrI	Mean CWHP_1_	Mean Half-Length of CrI	Coverage of 95% CrI	Mean CWHP_2_	Mean Half-Length of CrI
Hungary	100%	9%	5.1%	94%	17.8%	5.2%
Denmark	100%	5.5%	6.3%	100%	10.2%	6.5%
Southern Italy	100%	2.6%	3.8%	100%	7.1%	4.8%
Lombardy	100%	5.8%	6.9%	85%	2.4%	3.6%
Veneto	100%	3.8%	6.2%	71%	3.9%	5.1%
Chile	95%	9.2%	7.9%	100%	16.2%	9.5%

CWHP_1_ and CWHP_2_: conditional within-herd prevalence of primiparous and multiparous cows. CrI: credible interval for the prevalence estimate. Coverage: proportion of herds where the simulated infection probabilities of individual cows in parity subgroups fell within the CrI.

**Table 12:** Infection status of all 120 pseudo-herds (20 herds in each region) evaluated based on the Bayes factor, categorized according to Kass and Raftery [20].

Bayes Factor ^1^	Infection	Number of Truly Infected Herds	Number of Truly Not Infected Herds
0–0.05	strongly refuted	0	2
0.05–0.33	refuted	12	13
0.33–1	weakly refuted	18	8
1–3	weakly supported	15	1
3–20	supported	5	0
>20	strongly supported	46	0

^1^ not related to HTP prior.

**Table 13:** Infection status of all 120 pseudo-herds (20 herds in each region) evaluated based on the posterior probability of infection, categorized based on Kass and Raftery [20].

Posterior Probability ^1^	Infection	Number of Truly Infected Herds	Number of Truly Not Infected Herds
0–0.05	strongly refuted	0	0
0.05–0.25	refuted	1	4
0.25–0.5	weakly refuted	16	12
0.5–0.75	weakly supported	14	6
0.75–0.95	supported	12	2
0.95–1	strongly supported	53	0

^1^ incorporating HTP prior.

## Data Availability

The raw data supporting the conclusions of this article will be made available by the authors on request.

## Abbreviations

The following abbreviations are used in this manuscript:

AP	apparent prevalence
CrI	credible interval
CWHP	conditional within-herd prevalence
HTP	herd true prevalence
MAP	Mycobacterium avium subspecies paratuberculosis
MCMC	Markov Chain Monte Carlo
PTBC	bovine paratuberculosis
Se	sensitivity
Sp	specificity
TP	true prevalence

## Appendix A

**Table A1:** Information from the literature used to inform prior prevalence distributions of the model [8,10,12,23].

Region (Source of Prior Information)	Variable	Distribution from the Literature	Information
Denmark	HTP	beta (425.11, 144.64)	0.747 (0.781) ^a^
(posterior)	CWHP	beta (3.584, 45.398)	0.055 (0.16) ^b^
Southern Italy	HTP	beta (5.03, 7.04)	0.4 (0.2) ^b^
(prior)	CWHP	beta (μψ,(1 − μ)ψ)	−
	CWHP(μ)	beta (3.14, 31.7)	0.09 (0.18) ^c^
	CWHP(ψ)	gamma (11.16, 11.31)	−
Northern Italy (Lombardy, Veneto)	HTP	beta (13.32, 6.28)	0.70 (>0.50) ^b^
(prior)	CWHP	beta (μψ,(1 − μ)ψ)	−
	CWHP(μ)	beta (1.53, 15.69)	0.035 (<0.22) ^d^
	CWHP(ψ)	gamma (8.81, 1.42)	0.2 (<0.30) ^e^
Chile	HTP	beta (14.2, 0.7)	0.97 (0.99) ^e^
(prior)	CWHP	beta (μψ,(1 − μ)ψ)	−
	CWHP(μ)	beta (22.2, 176.9)	0.11 (0.15) ^e^
	CWHP(ψ)	gamma (9.1, 4.6)	0.25 (0.30) ^e^

HTP—herd true prevalence; CWHP—conditional within-herd prevalence; Se—sensitivity of ELISA test; Sp—specificity of ELISA test; ^a^—median and 95th percentile; ^b^—mode and 5th percentile; ^c^—estimates based on expert opinion: the mean prevalence is 0.09 with 95% certainty that it is not more than 0.18, where the expert is also confident that 90% of all herds have a prevalence less or equal to 0.35 with 95% certainty that it does not exceed 0.45; ^d^—mode and 95th percentile; ^e^—median and 95th percentile of the distribution of 90th percentile of the within-herd TP.

## Appendix B. Derivation of Priors for Conditional Within-Herd Prevalence of Subgroups Used in the Model

We collected historical information about the distribution of herd true prevalence (HTP) and within-herd true prevalence (CWHP) of PTBC infection from the literature to obtain informative priors. The priors from the studies representing different regions had the following form:

$$HTP\sim beta(\alpha_{HTP},\beta_{HTP}),$$

$$mCWHP\;\sim \;beta\left(\mu \psi ,\left(1-\mu \right)\psi \right),$$

$$\mu \sim beta\left(\alpha_{\mu },\;\beta_{\mu }\right),\;E\left(\mu \right)=\frac{\alpha_{\mu }}{\alpha_{\mu }+\beta_{\mu }},$$

$$\psi \sim gamma\left(\alpha_{\psi },\;\beta_{\psi }\right),\;E\left(\psi \right)=\alpha_{\psi }\times \beta_{\psi },$$

where mCWHP is the mean conditional within-herd prevalence, μ is the expected value, and ψ is the precision parameter of the distribution of mCWHP.

### Appendix B.1

The model requires the following priors:

- Herd true prevalence: $HTP\sim beta{(\alpha }_{HTP},\beta_{HTP})$.
- Mean conditional within-herd prevalence for primiparous cows: ${mCWHP}_{1}\sim beta\left(\mu_{1}\psi_{1},\left(1-\mu_{1}\right)\psi_{1}\right)$.
- Mean conditional within-herd prevalence for multiparous cows: ${mCWHP}_{2}\sim beta\left(\mu_{2}\psi_{2},\left(1-\mu_{2}\right)\psi_{2}\right)$.
- Variance of the herd random effect: $\sigma^{2}\sim inv.gamma\left(\alpha_{\sigma },\beta_{\sigma }\right).$
- Variance of the additive parity effect for primiparous cows: ${\sigma_{1}}^{2}\sim inv.gamma\left(\alpha_{\sigma_{1}},\beta_{\sigma_{1}}\right)$.
- Variance of the additive parity effect for multiparous cows: ${\sigma_{2}}^{2}\sim inv.gamma\left(\alpha_{\sigma_{2}},\beta_{\sigma_{2}}\right)$.

The $\mu_{1}$ and $\mu_{2}$ parameters of ${mCWHP}_{1}$ and $m{CWHP}_{2}$ priors were derived from the *mCWHP* prior using additional assumptions. Based on Hungarian data, the proportion of primiparous cows was assumed to be 40% and the ratio of $\mu_{1}$ and $\mu_{2}$ was assumed to be 2.

Assumptions:

- Population proportion of primiparous cows: p.
- Ratio of $\mu_{1}$ and $\mu_{2}$: R.
  (A1) $$E\left(\mu \right)=p\mu_{1}+\left(1-p\right)\mu_{2}$$
  (A2) $$\frac{\mu_{2}}{\mu_{1}}=R$$

From (A1) and (A2), we have $\mu_{1}$=$\frac{E(\mu )}{p\left(1-R\right)+R}$ and $\mu_{2}=\frac{R\times E(\mu )}{p\left(1-R\right)+R}$.

### Appendix B.2

The following priors were required for simulating the synthetic data:

- Conditional within-herd prevalence for primiparous cows: ${CWHP}_{1}\sim beta\left(\mu_{CWHP1}\psi_{CWHP1},\left(1-\mu_{CWHP1}\right)\psi_{CWHP1}\right)$
- Conditional within-herd prevalence for multiparous cows: ${CWHP}_{2}\sim beta\left(\mu_{CWHP2}\psi_{CWHP2},\left(1-\mu_{CWHP2}\right)\psi_{CWHP2}\right)$

$\mu_{CWHP1}$ and $\mu_{CWHP2}$ were chosen to be the mean $\mu_{1}$ and $\mu_{2}$ of ${mCWHP}_{1}\;$ and ${mCWHP}_{2}$ priors. To be coherent with the model, $\psi_{CWHP1}$ and $\psi_{CWHP2}$ were assigned the ψ parameters from the Hungarian study.

To model the joint infection probabilities of primiparous and multiparous cows within a herd, we used a Gaussian copula [27] with a correlation coefficient of ρ=0.89 [21]. The construction is the following: Let $Z_{1}\sim N\left(0,1\right),Z_{2}\sim N(0,1)$ with $Corr\left(Z_{1},Z_{2}\right)=0.89$. Let $U_{1}=\Phi \left(Z_{1}\right)$ and $U_{2}=\Phi \left(Z_{2}\right)$, where Φ is the cumulative standard normal distribution function. The joint distribution of the infection probabilities ${CWHP}_{1}$ and ${CWHP}_{2}$ of the parity groups is derived by transforming the uniformly distributed $U_{1}$ and $U_{2}$ using the inverse cumulative distribution functions of the respective prior distributions. Let $X_{1}=F_{{CWHP}_{1}}^{-1}\left(U_{1}\right)$ and $X_{2}=F_{{CWHP}_{2}}^{-1}\left(U_{2}\right)$, where $F_{{CWHP}_{.}}^{-1}$ is the inverse of the beta cumulative distribution function of CWHP. Thus,$\;(X_{1},X_{2})$ are random variables with the desired marginal distributions (that of ${CWHP}_{1}$ and ${CWHP}_{2}$) and a joint dependence structure induced via the Gaussian copula with ρ=0.89.

## Appendix C. Technical Description of the Single Herd Model

Let the parity be indexed by *j =* 1, 2 for primiparous and multiparous cows, respectively. Let Pos_jk_ be 1 if the *k*-th animal from the *j*-th parity is test positive and 0 if the selected animal is test negative. Further, let ***Pos_j_*** = (…, *Pos_j__k_*, …) be the vector containing all the *n_j_* test results for animals from the *j*-th parity group. We assume that the components of ***Pos_j_*** (indicating test results of all animals in parity group *j*) follow independent Bernoulli distributions.

(A3) $${Pos}_{j}\sim \prod_{k=1}^{n_{j}}Bernoulli\left({AP}_{j}\right),\;if\;the\;herd\;assumed\;to\;be\;infected,$$

(A4) $${Pos}_{j}\sim \prod_{k=1}^{n_{j}}Bernoulli\left(1-Sp\right),\;if\;the\;herd\;is\;assumed\;to\;be\;not\;infected.$$

${AP}_{j}$ represents the conditional within-herd apparent prevalence, which is the probability that a randomly selected individual from the *j*-th parity group tests positive, given the herd is infected. *Sp* is the specificity of the test. ${AP}_{j}$ can be expressed as the sum of the probability of true positive and false positive test results:

(A5) $${AP}_{j}=Se\times {CWHP}_{j}+\left(1-Sp\right)\times \left(1-{CWHP}_{j}\right),$$

where ${CWHP}_{j}$ is the conditional within-herd prevalence for the *j*-th parity group and Se, Sp stands for the sensitivity and specificity of the diagnostic test. From this point on, we assume that the herd is infected.

According to Veres et al. [21],

(A6) $${CHWP}_{Hj}=B_{\mu_{j},\psi_{j}}^{-1}\left(\Phi \left(\frac{b_{H}+b_{Hj}}{{\left(\sigma^{2}+\sigma_{j}^{2}\right)}^{\frac{1}{2}}}\right)\right),\;j=1,2\;\mathrm{and}$$

(A7) $$\psi_{j}=\frac{1}{\left(\sigma^{2}+\sigma_{j}^{2}\right)},$$

where $B^{-1}$ is the inverse of the beta cumulative distribution function.

We use region-specific priors for $\mu_{j}\;(j\;=\;1,2)$ and for the variances of the random effects ($\sigma^{2},\sigma_{j}^{2},\;j\;=\;1,2)$ in the following form:

$$\mu_{1}\;\sim \;beta\left(\alpha_{\mu_{1}},\beta_{\mu_{1}}\right),\;\mu_{2}\;\sim \;beta\left(\alpha_{\mu_{2}},\beta_{\mu_{2}}\right),$$

$$\sigma^{2}\sim inverse.gamma\left(\alpha_{\sigma },\;\beta_{\sigma }\right),\;{\sigma_{1}}^{2}\sim inverse.gamma\left(\alpha_{\sigma_{1}},\;\beta_{\sigma_{1}}\right),\;{\sigma_{2}}^{2}\sim inverse.gamma\left(\alpha_{\sigma_{2}},\;\beta_{\sigma_{2}}\right)$$

To compute posterior estimates of the ${CHWP}_{Hj},b_{H},and\;b_{Hj}$ parameters, four chains were run, each of 20,000 MCMC iterations. The runs were started from different sets of initial values of the parameters. The first 10,000 warm-up iterations in each chain were discarded. Convergence was checked by visual inspection of the trace plots of the chains. Additionally, the split R-hat formula [36] was used as convergence measure. Both approaches confirmed convergence.

The Bayesian model runs using the rstan package of the statistical software R 4.1.3 [30,31].
